# Clinical features and outcomes of patients with fever of unknown origin: a retrospective study

**DOI:** 10.1186/s12879-019-3834-5

**Published:** 2019-02-27

**Authors:** Yuting Tan, Xiaoqing Liu, Xiaochun Shi

**Affiliations:** 1 Department of Infectious Diseases, Peking Union Medical College Hospital, Chinese Academy of Medical Sciences and Peking Union Medical College, 1st Shuaifuyuan, Wangfujing, Dongcheng District, Beijing, 100730 China; 2 Centre for Tuberculosis Research, Chinese Academy of Medical Sciences and Peking Union Medical College, Beijing, 100730 China; 3 Clinical Epidemiology Unit, International Epidemiology Network, Chinese Academy of Medical Sciences and Peking Union Medical College, Beijing, 100730 China

**Keywords:** Fever of unknown origin, Follow-up, Diagnosis

## Abstract

**Background:**

Few studies have reported the long-term clinical outcome of patients discharged with undiagnosed fever of unknown origin (FUO). In this study, the clinical features and outcomes of patients with unexplained fever were explored to improve our understanding of FUO.

**Method:**

Patients diagnosed with FUO at admission and discharged without final diagnoses after systematic examination in the department of infectious diseases at Peking Union Medical College Hospital between 2004 and 2010 were followed up by telephone. Medical records were reviewed, and the clinical features and outcomes of patients for whom follow-up data were available were summarized.

**Results:**

Between 2004 and 2010, 58 patients with follow-up data, who were diagnosed with FUO at admission and did not have a final diagnosis at discharge, were enrolled in this study. The median duration of follow-up was 518 (0.4–830) weeks, and the fever duration was 24.6 (6.7–763.2) weeks. Final diagnoses were established in 11 cases (19%), and the diagnostic methods included clinical diagnosis, diagnostic therapy, genetic screening and biopsy pathology. The fever in 35 patients (60%) subsided during hospitalization or after discharge. Their condition was stable and self-limited after long-term follow-up, and they were ultimately thought to be cured. Two patients had periodic fever during prolonged observation: one patient needed intermittent use of nonsteroidal antiinflammatory drugs (NSAIDs), and the other needed intermittent use of NSAIDs and a steroid. Ten patients died during follow-up, with 9 deaths being caused by severe and worsening conditions related to the febrile illness.

**Conclusions:**

Long-term follow-up should be performed for patients with undiagnosed FUO. Some patients can obtain a definitive diagnosis by repeated multiple invasive examinations and diagnostic treatment. Most patients have a self-limited illness, and their prognosis is good.

## Background

In 1961, Petersdorf and Beeson formally proposed the definition of classic fever of unknown origin (FUO) by observing and summarizing a series of patients with unexplained fever as follows: a temperature > 38.3 °C on several occasions over a period of more than 3 weeks without a diagnosis despite 1 week of inpatient investigation [1]. At present, the aetiological classification of FUO includes infectious diseases, non-infectious inflammatory diseases, tumour diseases, other diseases and unknown diagnoses. Despite advances in medicine, the proportion of patients discharged with undiagnosed FUO after systematic examination has not decreased. Currently, the cause of febrile illness is not identified in approximately 9–51% of patients [2–5]. The reason may be that with the improvements in the quality of medical care and diagnostic tools, such as the popularization of advanced imaging technology, improvement in pathogen culture technology, newly developed serological detection projects, and application of polymerase chain reaction (PCR) technology, diagnosis rates of common diseases are improved. However, cases that meet the classic definition of FUO are becoming increasingly complex [5, 6]. Only a few studies to date have reported the outcome of patients who are discharged with undiagnosed FUO [2, 6–10]. In this study, patients who were diagnosed with FUO at admission and discharged without a final diagnosis in the department of infectious disease at Peking Union Medical College Hospital between 2004 and 2010 were followed for the purpose of investigating the clinical outcome of undiagnosed FUO.

## Methods

### Study population

Data on patients who were diagnosed with FUO at admission and discharged with undiagnosed FUO at our institution between 2004 and 2010 were collected, and those with available follow-up data were enrolled. FUO was diagnosed according to 1961 criteria [1].

### Methods

A retrospective and descriptive study was conducted. Patients who were diagnosed with FUO at admission and discharged at our institution between 2004 and 2010 were followed up by telephone to obtain their diagnosis and treatment after discharge. The medical records of patients with follow-up data were reviewed retrospectively. The clinical features and outcomes of these people were summarized.

### Auxiliary examination and treatment during hospitalization

**Fig. 1:**
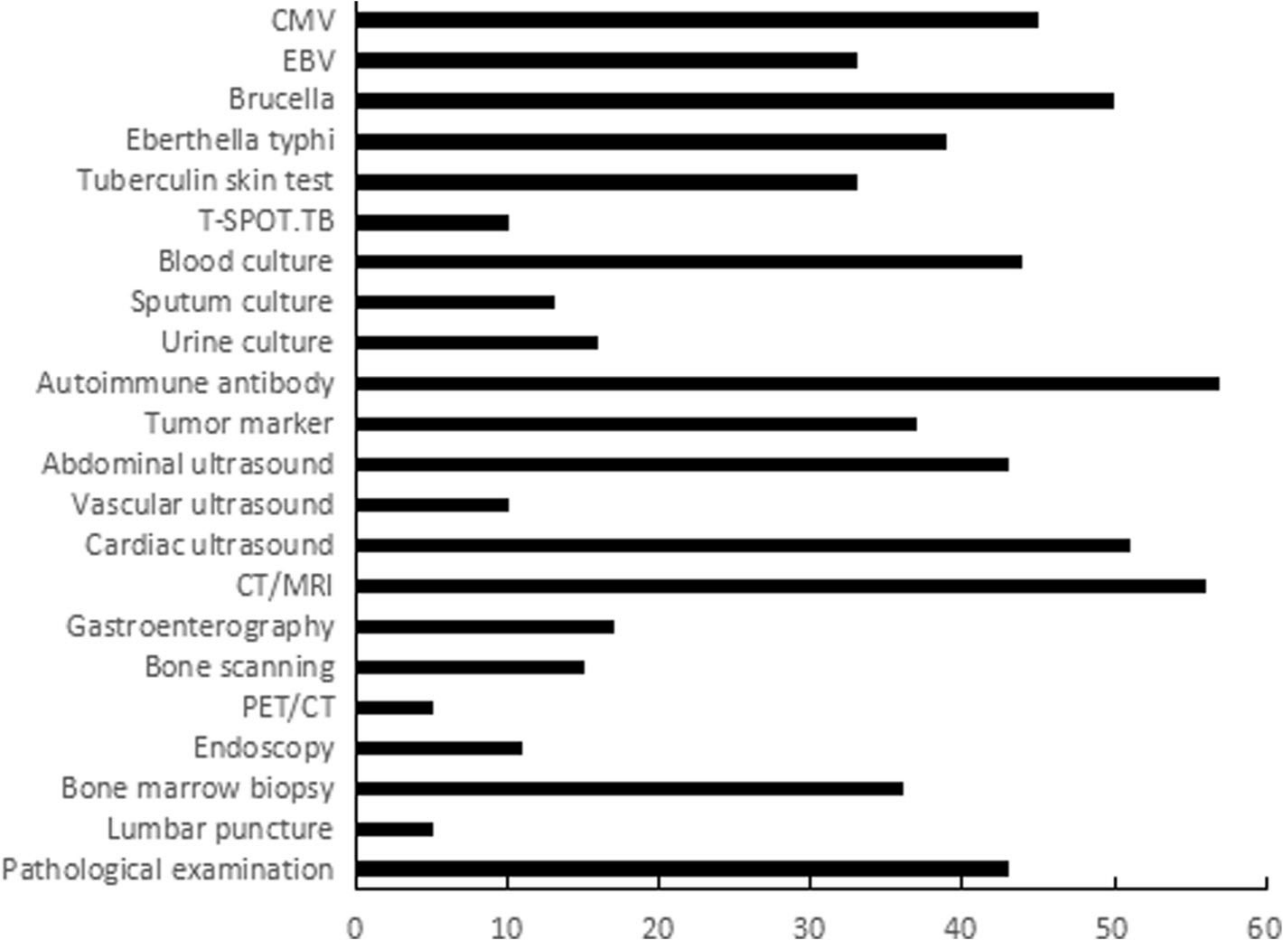
Auxiliary examination of 58 patients with FUO during hospitalization. CMV: Cytomegalovirus; EBV: Epstein-Barr virus; CT: computed tomography; and MRI: magnetic resonance imaging

Diagnostic clues were obtained from the detailed medical history and physical examination, and initial and specific examinations were performed. Some of the adjuvant examinations of the 58 patients during hospitalization are shown in Fig. 1. Overall, 74% of patients completed a pathological examination, including biopsy of bone marrow, lymph nodes, skin, pharynx mass, tonsil, bronchopulmonary tissue, liver, spleen and endometrium; however, no tumour-related pathological evidence was obtained. Thirty-six patients (62%) underwent bone marrow biopsy, and 52 bone marrow biopsies were performed. Seven patients with lymphadenopathy underwent 10 lymph node biopsies.

**Fig. 2:**
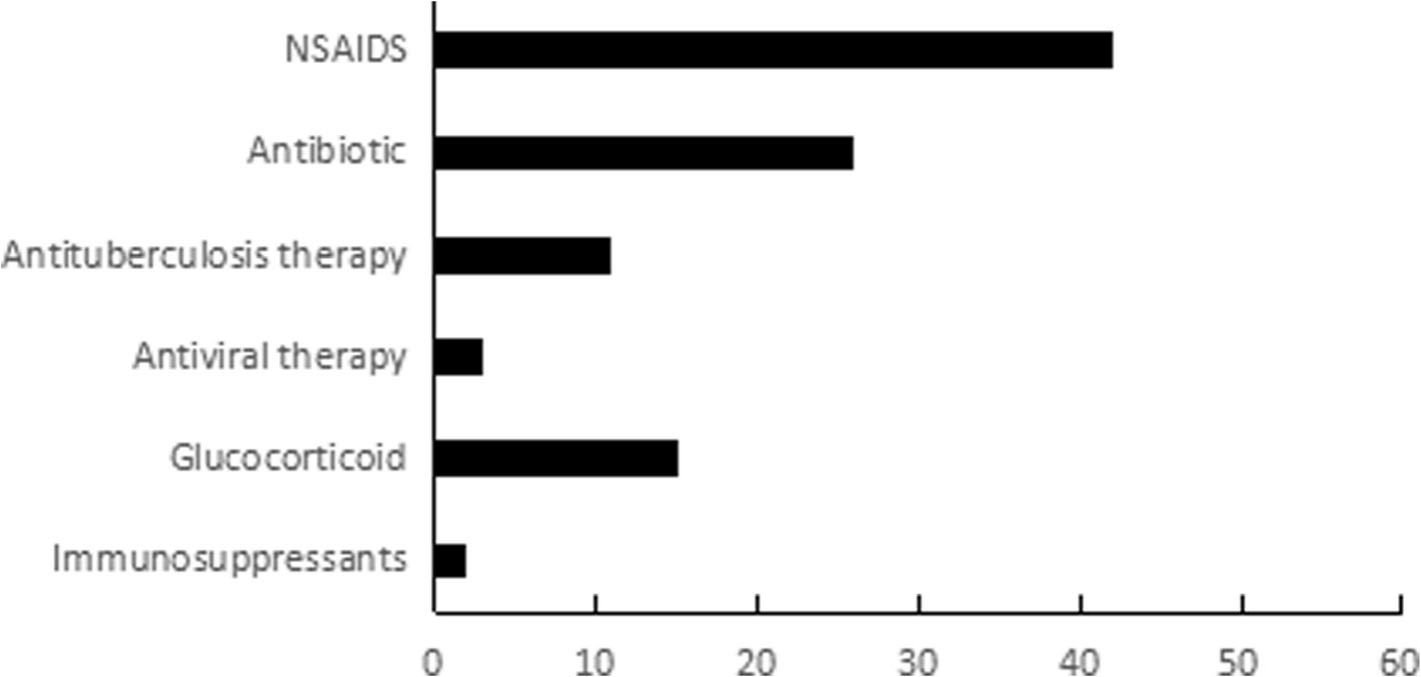
Treatment of 58 patients with FUO during hospitalization. NSAIDs: nonsteroidal antiinflammatory drugs

The treatment provided to the 58 patients during hospitalization is shown in Fig. 2. NSAIDs were given for treatment in 72% of cases (42/58). Considering that it was likely that most patients had an infectious disease, empirical antimicrobialswere given for treatment. Overall, 17% of patients (10/58) were given diagnostic anti-tuberculosis treatment. Approximately 26% of patients (15/58) were treated with steroids due to severe and worsening conditions. Two patients were considered to have adult Still’s disease (ASD) and received steroid and immunosuppressant treatment. Additionally, 20% of patients (12/58) did not receive any special treatment because of mild symptoms or a gradual decline in body temperature after admission.

### Statistical analysis

Data were collected and summarized using a standard form. Descriptive statistics were used to characterize the study population.

## Results

### Patient characteristics

Data for 200 patients diagnosed with FUO at admission and discharged with undiagnosed FUO at our institution between 2004 and 2010 were collected, with follow-up data available for 58 cases. The median age of the 58 patients was 39 years, ranging from 14 to 75 years. Twenty-eight patients (48%) were male, and 30 (52%) were female. Forty-nine (84%) were from urban areas, and 9 (16%) were from rural areas. The median duration of fever before admission was 12.9 (3–365) weeks, and the median duration of hospitalization was 3.8 (0.6–28) weeks. The clinical features of the 58 cases varied. Approximately 80% of patients had hyperpyrexia. The most common clinical features were weight loss (53%), hypoproteinaemia (52%), lymphadenectasis (52%), rash (48%), hepatosplenomegaly (40%), liver damage (36%), arthralgia (35%), and anaemia (20%). Details about other clinical characteristics are listed in Table 1.

**Table 1:** Demographic and clinical characteristics of 58 patients discharged with undiagnosed FUO

Variables	n (%)	Median (range)
Age (years)		39 (14–75)
Gender		
Male	28 (48)	
Female	30 (52)	
Residence		
Urban	49 (84)	
Rural	9 (16)	
Time of fever before admission (weeks)		12.9 (3–365)
Time of hospitalization (weeks)		3.8 (0.6–28)
Time from fever to discharge (weeks)		20.8 (6–369.6)
Tmax		
≤ 39 °C	11 (19)	
39–41 °C	46 (79)	
> 41 °C	1 (2)	
Disorder of consciousness	1 (2)	
Liver damage	21 (36)	
Anaemia	12 (20)	
Cytopenia	5 (8)	
Coagulation disorders	5 (8)	
Respiratory damage	7 (12)	
Gastrointestinal damage	3 (5)	
Renal function damage	1 (2)	

### Follow-up and clinical outcome

Definitive diagnoses were finally established in only 11 cases, and 6 of these were within 6 months after discharge (Table 2). The final diagnoses were tuberculosis, viral infection, autoinflammatory disease (AID), ASD and lymphoma. The diagnostic methods included clinical diagnosis, diagnostic therapy, gene screening and biopsy pathology. Clinical diagnosis was predominant in 5 cases, and the condition of these 5 patients was stable during the follow-up period. One patient was considered to have tuberculosis infection according to the epidemiological and clinical manifestations. He was finally diagnosed with tuberculosis due to the efficacy of diagnostic anti-tuberculosis treatment. Two patients were diagnosed with AID by gene screening, and 3 patients were diagnosed with lymphoma by biopsy pathology. They underwent 10 repeated invasive examinations during hospitalization, including bone marrow, lymph node, liver, pharyngeal mass, and tonsil biopsy, until pathological evidence was obtained. Of the 11 patients with a definitive diagnosis, the longest duration from febrile illness until diagnosis of disease was for AID at 621.1 weeks, followed by lymphoma (114.1 weeks).

**Table 2:** Final diagnoses of 11 FUO cases

	Final diagnosis	Time from febrile illness	Time from discharge	Diagnostic methods
		to diagnosis (weeks)	to diagnosis (weeks)	
1	Viral infection	7.4	1	Clinical diagnosis
2	Tuberculosis	64.5	23.9	Diagnostic therapy
3	AID	763.2	393.6	Genetic screening
4	AID	479.1	450.3	Genetic screening
5	ASD	38.3	17.3	Clinical diagnosis
6	ASD	46.7	23.9	Clinical diagnosis
7	ASD	53.4	5	Clinical diagnosis
8	ASD	86.2	46.6	Clinical diagnosis
9	Lymphoma	74.5	4.4	Pathology
10	Lymphoma	93.1	67.1	Pathology
11	Lymphoma	174.7	148.4	Pathology

The clinical outcomes of 58 patients discharged with FUO are shown in Table 3. Overall, 60% of patients (35/58) were ultimately cured but remained undiagnosed. The duration of fever before admission was 3–312.9 weeks. In 29 cases, fever subsided during hospitalization and after discharge. Two patients had repeated fever for 1 year and did not receive any treatment; 3 patients had periodic fever for 0.5–3 years and received NSAIDs for treatment; and one patient had periodic fever for 2 years and irregularly took oral steroid for treatment. Gastrointestinal bleeding, splenomegaly and hypersplenism appeared during his febrile period, and all complaints were resolved after splenectomy. All patients above were considered to have a self-limited clinical course and were ultimately cured. Two patients still had periodic fever during prolonged observation: one needed intermittent use of NSAIDs, and the other needed intermittent use of NSAIDs and a steroid. These two patients were generally in good condition. No progress and no drug-related complications occurred during prolonged observation.

**Table 3:** Outcomes after prolonged follow-up of 58 patients discharged with undiagnosed FUO

	n (%)
Final diagnosis	11 (19)
No final diagnosis	47 (81)
Spontaneous resolution	35 (60)
Recurring fever	2 (4)
Death	10 (17)

Ten patients died during the follow-up period, with a total mortality rate of 17.2%. Eight patients were male and 2 female. The median age of these patients was 54 (14–73) years. Nine of them died due to severe and worsening conditions related to the febrile illness. Five patients died within 1 month after discharge, two died after 0.5–1 year, and two died after 2–3 years. The last died after 10 years, and it was thus difficult to determine whether the cause of death was correlated to the febrile illness.

## Discussion

Since the definition of FUO was first proposed in 1961, FUO has remained a challenge in the diagnosis and treatment of difficult diseases. At present, few studies have reported the outcome of patients with undiagnosed FUO. The conclusions of these studies are consistent; that is, the majority of patients without a diagnosis after discharge have a good prognosis. Knockaert et al. [7] reported the outcome of 61 patients with undiagnosed FUO, and 41 patients were finally cured. Fever was relieved in 31 patients during hospitalization or shortly after discharge; 10 patients with prolonged fever for several months or years finally recovered and were considered to be cured. In Vanderschueren’s study [8], including 80 cases of undiagnosed FUO, 63 cases were finally cured but still undiagnosed. Zenone et al. [10] reported the outcome of 37 patients who were discharged with FUO, and 29 patients eventually recovered. In this study, 58 patients who were discharged with undiagnosed FUO at Peking Union Medical College Hospital were followed. Approximately 60% of patients recovered after discharge and were eventually considered to be cured.

For patients whose clinical manifestation is mild or relatively stable and whose aetiology is still unclear after detailed examination, long-term follow-up can be used in outpatient clinics to observe the disease duration. Both previous studies and our study have found that the clinical course of most FUO patients without diagnosis is self-limiting and that they are ultimately considered to be cured after long-term follow-up. For patients whose clinical manifestations may be in the early stages of the disease, follow-up and observation are essential means for obtaining the final diagnosis. In Li’s study [11], which included 70 cases of FUO, a patient with prolonged hyperpyrexia and liver damage was positive for anti-SSA antibody at 46 months of follow-up, and he was finally diagnosed with Sjogren’s syndrome.

It is important to search for the cause of disease in patients whose condition has deteriorated or has not been alleviated. Additional biopsies must be performed in difficult cases, especially when malignant diseases are suspected, to improve the positive rate of diagnosis. In a case series study [12] that included 105 cases of FUO, a total of 186 biopsies (including bone marrow, liver, lymph nodes) were performed in 86 patients. Finally, the diagnosis of 40 patients was confirmed by biopsy. Vanderschueren et al. [8] reported the diagnostic methods of 192 cases of FUO. Biopsy pathology was the most valuable diagnostic method. Approximately 24.5% of the patients (47/192) were diagnosed by biopsy.

Lymphoma is the most common malignant disease-causing FUO and accounts for 50–70% of cases [13, 14], and lymphoma with fever has always been a difficult condition to diagnose. Shi et al. [15] reported changes in the aetiological composition of FUO in our hospital from 1985 to 2010, with the proportion of lymphoma gradually increasing (from 36.4 to 68.4%). In 2003, Mao et al. [16] summarized the diagnostic methods of 50 cases of non-Hodgkin’s lymphoma with fever in our hospital. Multiple bone marrow biopsies were performed in all 50 patients, and a final diagnosis was established in 10 cases. Twenty-seven patients received multiple lymph node biopsies, and 14 cases received a definitive diagnosis. Of the 14 cases, one was diagnosed at the 7th lymph node biopsy, and pathological evidence was obtained from 6 patients by biopsy in two ways. In 2006, Li et al. [17] retrospectively analysed the clinical data from 53 cases of lymphoma with fever in our hospital. Fifty-three patients underwent at least 154 pathological examinations (including biopsy of bone marrow, superficial and deep lymph nodes, nasopharynx, subcutaneous nodules, liver, and spleen) before the final diagnosis was established. Nine cases were confirmed by multiple bone marrow biopsies; 44 biopsies of superficial or deep lymph nodes were conducted; and 13 patients had definitive diagnoses. Three patients were diagnosed by nasopharyngeal mass, 3 were diagnosed by subcutaneous nodules, and 25 were diagnosed by laparotomy. In our study, 3 patients diagnosed with lymphoma received 10 biopsies. A patient with prolonged hyperpyrexia, sore throat, purulent sinonasal mucus and swollen tonsils was diagnosed with lymphoma after multiple biopsies, including bone marrow, tonsil and two pharyngeal masses. A patient with hyperpyrexia, cough, lymphadenectasis, liver damage, blurred vision and hearing loss was finally diagnosed with non-Hodgkin’s lymphoma after biopsy of bone marrow, liver and pharynx.

In our study, 2 patients were diagnosed with AID. The duration from febrile illness until diagnosis was 9 and 15 years. The clinical manifestations included prolonged intermittent fever, rash, arthralgia, myalgia, splenomegaly and advanced inflammatory markers. No evidence of infection, tumours or other connective tissue diseases was obtained, and they were finally diagnosed with AID by genetic screening. AID is a group of hereditary non-invasive inflammatory diseases that are mainly caused by gene mutation in the coding protein, leading to inherent immune disorders and causing a systemic inflammatory response. AID is characterized by fever, rash, arthralgia, arthritis, and eye lesions and can affect multiple organ systems. Inflammatory markers, such as C-reactive protein (CRP) and erythrocyte sedimentation rate (ESR), are elevated, and no autoantibodies or only low titres of autoantibodies exist during disease flares [18]. AID is being increasingly recognized, and the genetic diagnosis of AID is being used more widely. However, the known mutation sites of AID-related genes still cannot be detected in approximately 60% of patients [19]. For patients whose clinical characteristics are consistent with AID and cannot be explained by infection or tumours, AID should be suspected, and genetic screening should be considered to assist diagnosis.

Clinical diagnoses can be performed when patients’ clinical characteristics are consistent with some specific diseases, the treatment is effective, no evidence of other diseases has been obtained after systematic examination, and the condition is stable after long-term follow-up. In this study, a patient with hyperpyrexia, headache, arthralgia, myalgia and lymphadenectasis had a 5-week febrile period before admission. Considering that he was more likely to have a viral infection according to the clinical manifestation and auxiliary examinations, NSAIDs were given for treatment, and his febrile illness was relieved. He had a self-limited clinical course (7.4 w) after long-term observation and was finally diagnosed with viral infection. Three patients whose clinical manifestations met ASD criteria demonstrated no evidence of infection or tumour after detailed examination. Glucocorticoid and antirheumatic drugs were effective, their condition was stable after prolonged follow-up, and they were finally diagnosed with ASD. In a retrospective study [20] of 153 patients with FUO, 53% of patients diagnosed with connective tissue diseases were clinically diagnosed. After exclusion of infections and tumours, these patients were eventually diagnosed with connective tissue disease based on their clinical features and response to diagnostic treatment.

In this study, only 19% of patients (11/58) received a definitive diagnosis after long-term follow-up. The cause may be that most patients without a final diagnosis have a good prognosis. Furthermore, the logistic regression method was used to evaluate whether age, sex, time of fever before admission, different system damage and the amount of damage were risk factors related to death in 58 patients discharged with undiagnosed FUO; however, positive results were not obtained. Then, 11 patients with definite diagnosis were removed, and whether the above factors were related to death was evaluated in 47 patients without a definite diagnosis; again, positive results were not obtained. The limited sample size may have resulted in the negative results.

Several limitations were observed in this study. First, this is a retrospective study with a small sample size in a single centre. Patients were followed by telephone, and more detailed information about diagnosis and treatment could not be obtained. Second, of the 200 patients discharged with undiagnosed FUO between 2004 and 2010, follow-up data were available for only 58. Thus, the results were not representative, and some bias occurred. The reason for the high rate of missing cases may be that our institution is a centre for complicated and severe diseases in China and that most of the patients are non-local residents. Patients in our study were enrolled between 2004 and 2010, and a complete follow-up model was not established at the time except for the telephone number. More than 10 years had passed and most of the patients or their families changed their contact information; thus, they could not be followed by telephone. Currently, only a few studies [2, 7–10] have reported the outcomes of patients discharged with undiagnosed FUO, and they have a small sample size and shorter follow-up duration (As shown in Table 4). In our study, the sample size was relatively large, and the information obtained was more accurate for each patient with follow-up data because of the long follow-up duration.

**Table 4:** Outcomes of patients discharged with undiagnosed FUO in different studies

Author/Year	Research type	Total Number of FUO^a^	Follow-up time	Number of undiagnosed cases	Number of patients with definite diagnosis	Number of patients with spontaneous resolution	Number of deaths
Knockaert, D. C. (1996) [7]	Retrospective study	61	5 years	49	12	41	6/2^b^
Vanderschueren S (2003) [8]	Prospective study	95	574 (125–2052) days	95	0	63	3
Zenone, T. (2006) [10]	Retrospective study	37	–	37	0	29	1
Bleeker-Rovers, C. P. (2007) [2]	Prospective study	37	12 (6–23) months	37	0	21	1
Mansueto, P. (2008) [9]	Retrospective study	29	2 years	21	8	13	4

^a^ Total number of patients discharged with undiagnosed FUO

^b^ Six patients died, although the cause of death was considered to be related to the disease that caused FUO in only two cases

## Conclusions

In conclusion, the illness is self-limited in most patients, and their prognosis is positive. Identifying the cause of disease in patients whose condition is deteriorated or not alleviated is important. In difficult diseases, multiple repeated invasive examinations are necessary to obtain a diagnosis.

## Abbreviations

AID: Autoinflammatory disease
ANA: Antinuclear antibodies
ANCA: Antineutrophil cytoplasmic antibodies
ASD: Adult Still’s disease
CMV: Cytomegalovirus
CT: Computed tomography
EBV: Epstein-Barr virus
ENA: Extractable nuclear antigens
FUO: Fever of unknown origin
MRI: Magnetic resonance imaging
NSAIDs: Nonsteroidal antiinflammatory drugs
PCR: Polymerase chain reaction
